# Crystal structure of 6,7-dihy­droxy-6,7-di­hydro-3*H*-imidazo[1,2-*a*]purin-9(5*H*)-one

**DOI:** 10.1107/S2056989016009087

**Published:** 2016-07-19

**Authors:** Wei Guo, Cheng-Xun Li, Jie Lv, Jing Wang

**Affiliations:** a School of Pharmacy, Hebei Medical University, Shijiazhuang 050017, People’s Republic of China

**Keywords:** crystal structure, purine derivative, hydrogen bonding, framework structure

## Abstract

The title purine derivative, C_7_H_7_N_5_O_3_, is an adduct of guanine with glyoxal. In the mol­ecule, the di­hydro­imidazole ring adopts a twisted conformation on the C—C bond, and the two hydroxyl groups lie on opposite sides of the mean plane of the ring. In the crystal, the mol­ecules are linked by N—H⋯O, O—H⋯N and N—H⋯N hydrogen bonds forming a three-dimensional framework. The crystal packing is reinforced by C—H⋯O hydrogen bonds and by offset π–π stacking of the purine ring systems of inversion related mol­ecules [inter­centroid distance = 3.4839 (12) Å].

## Abstract 2

In the crystal of the title purine derivative, mol­ecules are linked by O—H⋯N, N—H⋯O and N—H⋯N hydrogen bonds, forming a three-dimensional framework.

## Chemical context   

Purine are essential ingredients of various compounds, for example two of the five bases in nucleic acids, adenine and guanine, are purines. Purine derivatives have been developed as inhibitors of cyclin-dependent kinase (Sausville, 2002 ▸), and as anti­parasitic (Braga *et al.*, 2007 ▸; Yadav *et al.*, 2004 ▸), anti­tumor (Prekupec *et al.*, 2003 ▸; Trávníček *et al.*, 2001 ▸), anti­radical (Klanicová *et al.*, 2010 ▸) and anti­viral (Manikowski *et al.*, 2005 ▸) drugs. The synthesis and the cancerostatic and anti­viral activities of the title compound were reported on many years ago (Shapiro *et al.*, 1969 ▸). Its crystal structure has not been reported to date, and as the conformation of a biologically active mol­ecule is crucial to its activity we undertook the structure analysis of the title compound, which we report on herein.
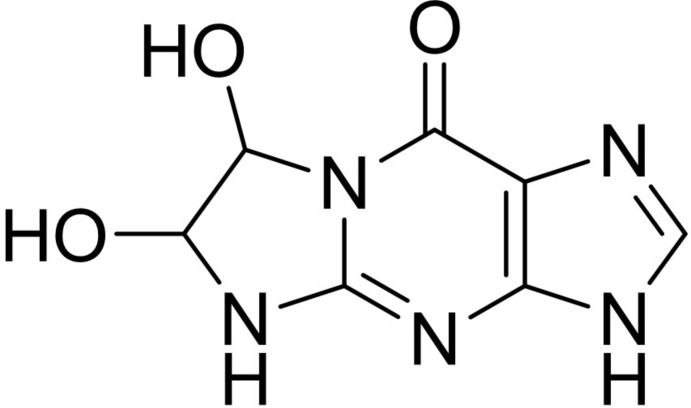


## Structural commentary   

**Figure 1:**
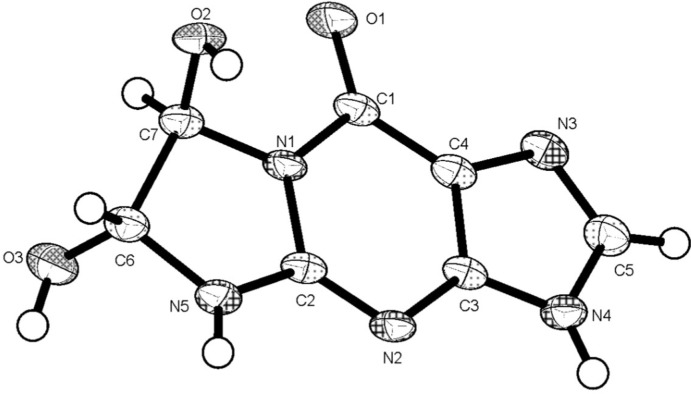
The mol­ecular structure of the title compound, with atom labelling. Displacement ellipsoids are drawn at the 50% probability level.

The mol­ecular structure of title compound is depicted in Fig. 1 ▸. The C1—O1 bond length of 1.220 (2) Å shows typical double-bond character, and is coplanar with the purine moiety for their aromatic nature. The non-aromatic five-membered ring (N1/C7/C6/N5/C2) adopts a twisted conformation on the C6—C7 bond. The two hydroxyl groups lie on opposite sides of the ring mean plane with an O2—C7—C6—O3 torsion angle of 114.8 (2)°.

## Supra­molecular features   

**Figure 2:**
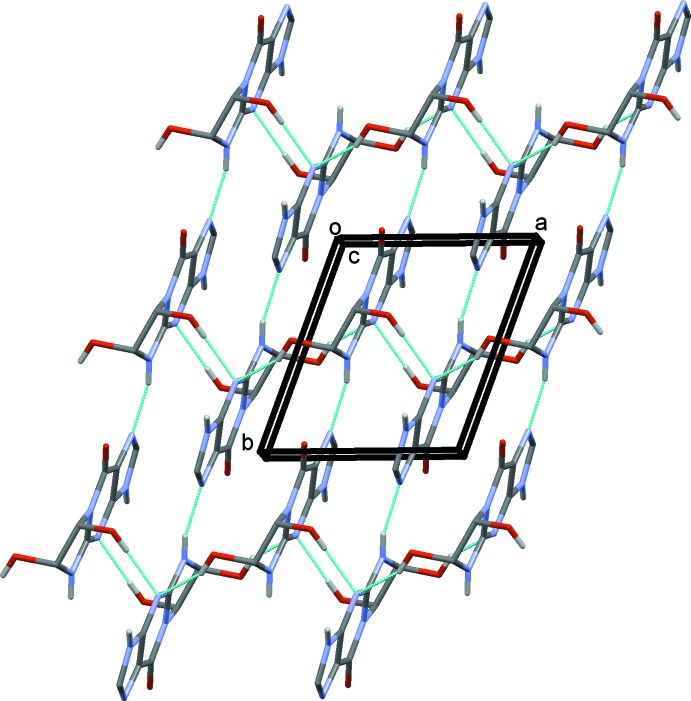
A view along the *c* axis of the crystal packing of the title compound. The hydrogen bonds are shown as dashed lines (see Table 1 ▸).

**Figure 3:**
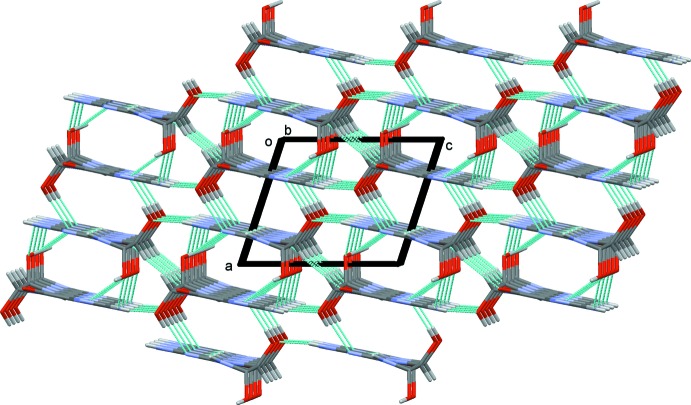
A view along the *b* axis of the crystal packing of the title compound. The hydrogen bonds are shown as dashed lines (see Table 1 ▸).

In the crystal, mol­ecules are also linked *via* O—H⋯N and N—H⋯O hydrogen bonds, forming layers lying parallel to the *ab* plane (Table 1 ▸ and Fig. 2 ▸). The layers are linked by N—H⋯O hydrogen bonds, forming a three-dimensional framework (Table 1 ▸ and Fig. 3 ▸). Within the framework there are also C—H⋯O hydrogen bonds present (Table 1 ▸), and inversion-related mol­ecules are linked by offset π–π inter­actions involving the five-membered ring and the six-membered ring of the purine moieties [*Cg*2⋯*Cg*3^i^ = 3.4839 (12) Å, inter­planar distance = 3.311 (1) Å, slippage = 1.112 Å; *Cg*2 and *Cg*3 are the centroids of the N3/C4/C3/N4/C5 and N1/C1/C4/C3/N2/C2 rings, respectively; symmetry code: (i) −*x*, −*y*, −*z* + 2].

## Database survey   

A search of the Cambridge Structural Database (CSD, Version 5.37, update May 2016; Groom *et al.*, 2016 ▸) for 1,9-di­hydro-6*H*-6-one as substructure, gave 61 hits. Many of these compounds concern guanine and guaninium and some metal complexes, but none involve a fused third ring. The structure of the title compound has not been reported previously.

## Synthesis and crystallization   

The title compound was synthesized according to a literature method (Dey & Garner, 2000 ▸): An aqueous solution (1 l) of glyoxal monohydrate (8.71 g, 0.18 mol), guanine (1.55g, 0.01 mol) and a small amount of acetic acid was stirred for 24 h at 333 K. Then the excess water was removed by rotary evaporation and 250 ml of THF was added under stirring. The white suspension that formed was suction-filtered and washed with THF. The product was obtained as white powder after drying at 313 K. Colourless block-shaped crystals suitable for X-ray diffraction were obtained by recrystallization of the powder in a DMF/ethanol/water (*v*/*v*/*v* = 1/2/2) medium.

## Refinement   

Crystal data, data collection and structure refinement details are summarized in Table 2 ▸. All of the H atoms were positioned with idealized geometry and refined as riding: O—H = 0.82 Å, N—H = 0.86 Å and C—H = 0.93–0.98 Å, with *U*_iso_(H) = 1.2*U*_eq_(C) and 1.5*U*_eq_(O,N).

## Supplementary Material

Crystal structure: contains datablock(s) I, global. DOI: 10.1107/S2056989016009087/xu5887sup1.cif

Structure factors: contains datablock(s) I. DOI: 10.1107/S2056989016009087/xu5887Isup2.hkl

Supporting information file. DOI: 10.1107/S2056989016009087/xu5887Isup3.cml

CCDC reference: 1469976

Additional supporting information: crystallographic information; 3D view; checkCIF report

## Figures and Tables

**Table 1:** Hydrogen-bond geometry (Å, °)

*D*—H⋯*A*	*D*—H	H⋯*A*	*D*⋯*A*	*D*—H⋯*A*
O2—H2⋯N2^i^	0.82	2.03	2.850 (2)	178
O3—H3⋯N2^ii^	0.82	2.21	2.947 (2)	150
N4—H4⋯O2^iii^	0.86	1.95	2.791 (2)	167
N5—H5⋯N3^iv^	0.86	2.00	2.837 (2)	166
C5—H5*A*⋯O3^v^	0.93	2.50	3.133 (8)	126
C7—H7⋯O1^vi^	0.98	2.51	3.449 (2)	161

Symmetry codes: (i) 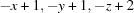; (ii) 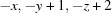; (iii) 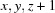; (iv) 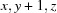; (v) 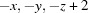; (vi) 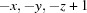.

**Table 2:** Experimental details

Crystal data	
Chemical formula	C_7_H_7_N_5_O_3_
*M*_r_	209.18
Crystal system, space group	Triclinic, *P* 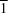
Temperature (K)	295
*a*, *b*, *c* (Å)	6.8925 (4), 7.6352 (4), 8.0605 (6)
α, β, γ (°)	95.063 (5), 105.135 (6), 107.647 (5)
*V* (Å^3^)	383.69 (4)
*Z*	2
Radiation type	Cu *K*α
μ (mm^−1^)	1.26
Crystal size (mm)	0.20 × 0.18 × 0.16

Data collection	
Diffractometer	Agilent Xcalibur, Eos, Gemini
Absorption correction	Multi-scan (*CrysAlis PRO*; Agilent, 2012 ▸)
*T*_min_, *T*_max_	0.52, 0.82
No. of measured, independent and observed [*I* > 2σ(*I*)] reflections	2506, 1499, 1335
*R*_int_	0.033
(sin θ/λ)_max_ (Å^−1^)	0.622

Refinement	
*R*[*F* ^2^ > 2σ(*F* ^2^)], *wR*(*F* ^2^), *S*	0.057, 0.180, 1.00
No. of reflections	1499
No. of parameters	138
H-atom treatment	H-atom parameters constrained
Δρ_max_, Δρ_min_ (e Å^−3^)	0.67, −0.40

Computer programs: *CrysAlis PRO* (Agilent, 2012 ▸), *SHELXS97*, *SHELXL97* and *SHELXTL* (Sheldrick, 2008 ▸) and *PLATON* (Spek, 2009 ▸).

## supplementary crystallographic information

### Crystal data

**Table:**

C_7_H_7_N_5_O_3_	*Z* = 2
*M**_r_* = 209.18	*F*(000) = 216
Triclinic, *P*1	*D*_x_ = 1.811 Mg m^−^^3^
Hall symbol: -P 1	Cu *K*α radiation, λ = 1.54178 Å
*a* = 6.8925 (4) Å	Cell parameters from 1705 reflections
*b* = 7.6352 (4) Å	θ = 7.0–73.5°
*c* = 8.0605 (6) Å	µ = 1.26 mm^−^^1^
α = 95.063 (5)°	*T* = 295 K
β = 105.135 (6)°	Block, colorless
γ = 107.647 (5)°	0.20 × 0.18 × 0.16 mm
*V* = 383.69 (4) Å^3^	

### Data collection

**Table:**

Agilent Xcalibur, Eos, Gemini diffractometer	1499 independent reflections
Radiation source: Enhance (Cu) X-ray Source	1335 reflections with *I* > 2σ(*I*)
Graphite monochromator	*R*_int_ = 0.033
Detector resolution: 5.3031 pixels mm^-1^	θ_max_ = 73.7°, θ_min_ = 5.8°
ω scans	*h* = −8→8
Absorption correction: multi-scan (CrysAlis PRO; Agilent, 2012)	*k* = −9→7
*T*_min_ = 0.52, *T*_max_ = 0.82	*l* = −10→9
2506 measured reflections	

### Refinement

**Table:**

Refinement on *F*^2^	Primary atom site location: structure-invariant direct methods
Least-squares matrix: full	Secondary atom site location: difference Fourier map
*R*[*F*^2^ > 2σ(*F*^2^)] = 0.057	Hydrogen site location: inferred from neighbouring sites
*wR*(*F*^2^) = 0.180	H-atom parameters constrained
*S* = 1.00	*w* = 1/[σ^2^(*F*_o_^2^) + (0.1404*P*)^2^ + 0.079*P*] where *P* = (*F*_o_^2^ + 2*F*_c_^2^)/3
1499 reflections	(Δ/σ)_max_ < 0.001
138 parameters	Δρ_max_ = 0.67 e Å^−^^3^
0 restraints	Δρ_min_ = −0.40 e Å^−^^3^

### Special details

**Table:**

Experimental. CrysAlis Pro, Agilent Technologies, Version 1.171.35.21 (release 20-01-2012 CrysAlis171 .NET) (compiled Jan 23 2012,18:06:46) Empirical absorption correction using spherical harmonics, implemented in SCALE3 ABSPACK scaling algorithm.
Geometry. All esds (except the esd in the dihedral angle between two l.s. planes) are estimated using the full covariance matrix. The cell esds are taken into account individually in the estimation of esds in distances, angles and torsion angles; correlations between esds in cell parameters are only used when they are defined by crystal symmetry. An approximate (isotropic) treatment of cell esds is used for estimating esds involving l.s. planes.
Refinement. Refinement of F^2^ against ALL reflections. The weighted R-factor wR and goodness of fit S are based on F^2^, conventional R-factors R are based on F, with F set to zero for negative F^2^. The threshold expression of F^2^ > 2sigma(F^2^) is used only for calculating R-factors(gt) etc. and is not relevant to the choice of reflections for refinement. R-factors based on F^2^ are statistically about twice as large as those based on F, and R- factors based on ALL data will be even larger.

### Fractional atomic coordinates and isotropic or equivalent isotropic displacement parameters (Å^2^)

**Table:**

	*x*	*y*	*z*	*U*_iso_*/*U*_eq_	
O1	0.1803 (3)	−0.0561 (2)	0.71668 (19)	0.0330 (4)	
O2	0.3726 (2)	0.34594 (19)	0.61681 (18)	0.0278 (4)	
H2	0.4725	0.4310	0.6862	0.042*	
O3	−0.0753 (2)	0.4579 (2)	0.6656 (2)	0.0355 (4)	
H3	−0.1043	0.5434	0.7105	0.053*	
N1	0.2147 (2)	0.2403 (2)	0.8339 (2)	0.0222 (4)	
N2	0.2866 (3)	0.3571 (2)	1.1379 (2)	0.0234 (4)	
N3	0.2741 (3)	−0.1191 (2)	1.1004 (2)	0.0267 (4)	
N4	0.3297 (3)	0.1187 (2)	1.3093 (2)	0.0254 (4)	
H4	0.3563	0.1802	1.4119	0.031*	
N5	0.2310 (3)	0.5316 (2)	0.9134 (2)	0.0278 (4)	
H5	0.2665	0.6384	0.9789	0.033*	
C1	0.2159 (3)	0.0573 (2)	0.8471 (3)	0.0227 (5)	
C2	0.2459 (3)	0.3764 (2)	0.9729 (2)	0.0217 (5)	
C3	0.2944 (3)	0.1830 (2)	1.1558 (2)	0.0212 (4)	
C4	0.2612 (3)	0.0354 (2)	1.0264 (3)	0.0226 (5)	
C5	0.3142 (3)	−0.0636 (3)	1.2673 (3)	0.0277 (5)	
H5A	0.3306	−0.1402	1.3496	0.033*	
C6	0.1474 (3)	0.4966 (3)	0.7242 (3)	0.0245 (5)	
H6	0.2227	0.5999	0.6752	0.029*	
C7	0.1903 (3)	0.3160 (3)	0.6712 (2)	0.0232 (5)	
H7	0.0653	0.2300	0.5793	0.028*	

### Atomic displacement parameters (Å^2^)

**Table:**

	*U*^11^	*U*^22^	*U*^33^	*U*^12^	*U*^13^	*U*^23^
O1	0.0427 (8)	0.0254 (8)	0.0294 (8)	0.0170 (6)	0.0067 (6)	−0.0085 (6)
O2	0.0276 (7)	0.0275 (7)	0.0277 (7)	0.0082 (6)	0.0123 (6)	−0.0061 (5)
O3	0.0370 (8)	0.0364 (9)	0.0395 (9)	0.0203 (7)	0.0142 (7)	0.0028 (7)
N1	0.0276 (8)	0.0173 (8)	0.0235 (8)	0.0109 (6)	0.0088 (6)	−0.0023 (6)
N2	0.0298 (8)	0.0183 (8)	0.0239 (8)	0.0102 (6)	0.0106 (7)	−0.0020 (6)
N3	0.0307 (8)	0.0173 (8)	0.0331 (9)	0.0099 (6)	0.0106 (7)	0.0008 (6)
N4	0.0315 (8)	0.0225 (8)	0.0242 (8)	0.0107 (7)	0.0112 (6)	0.0002 (6)
N5	0.0421 (10)	0.0175 (8)	0.0264 (8)	0.0148 (7)	0.0109 (7)	−0.0016 (6)
C1	0.0224 (8)	0.0172 (9)	0.0276 (10)	0.0078 (7)	0.0073 (7)	−0.0038 (7)
C2	0.0233 (8)	0.0172 (8)	0.0248 (9)	0.0071 (7)	0.0093 (7)	−0.0027 (7)
C3	0.0213 (8)	0.0160 (9)	0.0269 (9)	0.0069 (6)	0.0095 (7)	−0.0007 (7)
C4	0.0233 (8)	0.0162 (8)	0.0281 (10)	0.0073 (7)	0.0086 (7)	−0.0014 (7)
C5	0.0323 (10)	0.0212 (10)	0.0313 (10)	0.0115 (8)	0.0102 (8)	0.0025 (7)
C6	0.0310 (9)	0.0201 (9)	0.0270 (10)	0.0117 (7)	0.0131 (8)	0.0033 (7)
C7	0.0259 (9)	0.0202 (9)	0.0237 (9)	0.0088 (7)	0.0084 (7)	−0.0018 (7)

### Geometric parameters (Å, º)

**Table:**

O1—C1	1.221 (2)	N4—C3	1.361 (3)
O2—C7	1.398 (2)	N4—C5	1.367 (2)
O2—H2	0.8200	N4—H4	0.8600
O3—C6	1.410 (2)	N5—C2	1.339 (3)
O3—H3	0.8200	N5—C6	1.450 (2)
N1—C2	1.384 (2)	N5—H5	0.8600
N1—C1	1.413 (2)	C1—C4	1.433 (3)
N1—C7	1.470 (2)	C3—C4	1.386 (2)
N2—C2	1.316 (2)	C5—H5A	0.9300
N2—C3	1.365 (2)	C6—C7	1.543 (2)
N3—C5	1.304 (3)	C6—H6	0.9800
N3—C4	1.384 (2)	C7—H7	0.9800
			
C7—O2—H2	109.5	N4—C3—C4	106.17 (16)
C6—O3—H3	109.5	N2—C3—C4	128.41 (18)
C2—N1—C1	125.05 (16)	N3—C4—C3	109.67 (17)
C2—N1—C7	110.23 (15)	N3—C4—C1	130.23 (17)
C1—N1—C7	124.65 (15)	C3—C4—C1	120.08 (17)
C2—N2—C3	111.14 (15)	N3—C5—N4	113.27 (18)
C5—N3—C4	104.69 (16)	N3—C5—H5A	123.4
C3—N4—C5	106.20 (16)	N4—C5—H5A	123.4
C3—N4—H4	126.9	O3—C6—N5	112.28 (16)
C5—N4—H4	126.9	O3—C6—C7	107.73 (15)
C2—N5—C6	111.39 (14)	N5—C6—C7	102.64 (14)
C2—N5—H5	124.3	O3—C6—H6	111.3
C6—N5—H5	124.3	N5—C6—H6	111.3
O1—C1—N1	120.75 (18)	C7—C6—H6	111.3
O1—C1—C4	129.30 (18)	O2—C7—N1	111.34 (15)
N1—C1—C4	109.95 (15)	O2—C7—C6	114.01 (15)
N2—C2—N5	125.43 (16)	N1—C7—C6	101.82 (14)
N2—C2—N1	125.33 (17)	O2—C7—H7	109.8
N5—C2—N1	109.23 (16)	N1—C7—H7	109.8
N4—C3—N2	125.38 (16)	C6—C7—H7	109.8
			
C2—N1—C1—O1	177.72 (17)	N2—C3—C4—N3	−177.14 (17)
C7—N1—C1—O1	−5.4 (3)	N4—C3—C4—C1	179.03 (16)
C2—N1—C1—C4	−1.9 (2)	N2—C3—C4—C1	1.3 (3)
C7—N1—C1—C4	175.00 (15)	O1—C1—C4—N3	−1.0 (3)
C3—N2—C2—N5	−179.08 (18)	N1—C1—C4—N3	178.54 (17)
C3—N2—C2—N1	−0.1 (3)	O1—C1—C4—C3	−179.13 (19)
C6—N5—C2—N2	−169.71 (17)	N1—C1—C4—C3	0.4 (2)
C6—N5—C2—N1	11.1 (2)	C4—N3—C5—N4	−0.3 (2)
C1—N1—C2—N2	1.8 (3)	C3—N4—C5—N3	0.6 (2)
C7—N1—C2—N2	−175.41 (17)	C2—N5—C6—O3	95.18 (19)
C1—N1—C2—N5	−179.00 (16)	C2—N5—C6—C7	−20.2 (2)
C7—N1—C2—N5	3.7 (2)	C2—N1—C7—O2	106.35 (17)
C5—N4—C3—N2	177.11 (17)	C1—N1—C7—O2	−70.9 (2)
C5—N4—C3—C4	−0.7 (2)	C2—N1—C7—C6	−15.51 (18)
C2—N2—C3—N4	−178.80 (17)	C1—N1—C7—C6	167.22 (16)
C2—N2—C3—C4	−1.5 (3)	O3—C6—C7—O2	141.81 (16)
C5—N3—C4—C3	−0.2 (2)	N5—C6—C7—O2	−99.53 (18)
C5—N3—C4—C1	−178.47 (19)	O3—C6—C7—N1	−98.19 (17)
N4—C3—C4—N3	0.6 (2)	N5—C6—C7—N1	20.48 (18)

### Hydrogen-bond geometry (Å, º)

**Table:**

*D*—H···*A*	*D*—H	H···*A*	*D*···*A*	*D*—H···*A*
O2—H2···N2^i^	0.82	2.03	2.850 (2)	178
O3—H3···N2^ii^	0.82	2.21	2.947 (2)	150
N4—H4···O2^iii^	0.86	1.95	2.791 (2)	167
N5—H5···N3^iv^	0.86	2.00	2.837 (2)	166
C5—H5*A*···O3^v^	0.93	2.50	3.133 (8)	126
C7—H7···O1^vi^	0.98	2.51	3.449 (2)	161

Symmetry codes: (i) −*x*+1, −*y*+1, −*z*+2; (ii) −*x*, −*y*+1, −*z*+2; (iii) *x*, *y*, *z*+1; (iv) *x*, *y*+1, *z*; (v) −*x*, −*y*, −*z*+2; (vi) −*x*, −*y*, −*z*+1.
